# Enterotoxigenic Potential of Coagulase-Negative Staphylococci from Ready-to-Eat Food

**DOI:** 10.3390/pathogens9090734

**Published:** 2020-09-06

**Authors:** Wioleta Chajęcka-Wierzchowska, Joanna Gajewska, Patryk Wiśniewski, Anna Zadernowska

**Affiliations:** Department of Industrial and Food Microbiology, University of Warmia and Mazury in Olsztyn, Plac Cieszyński 1, 10-726 Olsztyn, Poland; joanna.gajewska@uwm.edu.pl (J.G.); patryk.wisniewski.2@student.uwm.edu.pl (P.W.); anna.zadernowska@uwm.edu.pl (A.Z.)

**Keywords:** coagulase-negative staphylococci, ready-to-eat food, staphylococcal enterotoxins

## Abstract

Although coagulase-positive staphylococci are considered to be the main factor responsible for food poisoning, an increasing role for the coagulase-negative staphylococci in the production of enterotoxins has been observed in recent years. This study was conducted to assess the occurrence of genes responsible for the production of staphylococcal enterotoxins (SE), enterotoxin-like toxins (SEI) and toxic shock syndrome toxin-1 (TSST-1) in coagulase-negative staphylococci (CoNS) isolated from ready-to-eat food from bars and restaurants. One hundred and eighteen CoNS strains were tested using polymerase chain reaction (PCR) to five superantigenic toxin genes, including five different types of classical enterotoxins (*sea, seb, sec, sed* and *see*) and the toxic shock syndrome toxin-1 (*tsst*-1) as well as to supertoxin-like genes. PCR-positive isolates were then tested using immunoenzymatic methods (SET-RPLA, Vidas SET 2) for toxin expression. Out of 118 CoNS strains, the presence of staphylococcal enterotoxins was confirmed in 72% of them. The most frequently found enterotoxin-like genotype was *ser*, *selu*. Two of the tested strains had up to ten different enterotoxin genes in the genome at the same time. Although no production of enterotoxins was detected in the CoNS, which means that their possible role in the epidemiology of food-borne diseases is minimal, the data demonstrated that the toxigenic capacity of the CoNS should not be ignored, and that this group of microorganisms should be continuously monitored in food.

## 1. Introduction

Coagulase-negative staphylococci (CoNS) are among the strains most commonly found in food, including ready-to-eat products. They are present in food far more frequently than coagulase-positive staphylococci (CPS). In Europe, however, it is not required to determine their presence in food. Pursuant to the Commission Regulation (EC) No 2073/2005 of 15 November 2005 [1], as subsequently amended (on microbiological criteria for foodstuffs), only the determination of coagulase-positive staphylococci (CPS) is included in the hygiene criteria. Considering that CoNS are very commonly found in food, it is suggested that they may play an important role in the epidemiology of food-borne diseases.

Staphylococcal Foodborne Poisoning (SFP) is among the most common food-borne diseases. For years, it was believed that only coagulase-positive *Staphylococcus aureus* strains had the ability to produce enterotoxins and induce food poisoning. On the other hand, coagulase-negative staphylococcal (CoNS) strains for years were considered to be non-pathogenic to humans and were regarded as microflora incapable of inducing infection or poisoning. Interest in these strains has been growing significantly as a result of the increasing role they play in causing infections in humans due to their increasing resistance to antibiotics [2]. This also makes them an interesting object of research into the possibilities for inducing food poisoning due to their ability to produce enterotoxins. Studies conducted on clinical strains have provided evidence suggesting that certain CoNS strains may be major hospital pathogens [3,4]. These include strains capable of producing superantigenic toxins such as staphylococcal enterotoxins (SE), toxic shock syndrome toxin-1 (TSST-1) and other toxins, including hemolysins, coagulase and exfoliative toxins [5].

According to the data of the European Food Safety Authority and the European Centre for Disease Control (EFSA and ECDC), bacterial toxins are the third major causative agent group in Europe. Staphylococcal enterotoxins (SE) are responsible for half of the reported cases of announced epidemics caused by bacterial toxins. It should also be noted that in cases of enterotoxin poisoning occurrence, it is extremely difficult to determine whether the enterotoxin was produced by coagulase-negative or positive strains (especially in food after thermal treatment like pasteurisation, sterilization, scalding or cooking). This is because tests detect the enterotoxin itself, without checking for the presence of viable staphylococcal cells. Therefore, the involvement of CoNS in inducing food poisoning associated with the production of toxins is possible.

Staphylococci capable of producing enterotoxins pose a significant public health problem, as food processing operations do not deactivate toxins. In contrast to the bacteria themselves, enterotoxins are characterised by increased resistance to high temperatures, a wide pH range and proteolytic enzymes [6]. They are also resistant to such procedures as freezing or drying and are insensitive to enzymatic digestion in the human digestive tract [7]. Most cases of food poisoning are due to poor hygiene practice during the production process and product distribution [8]. Following contamination, maintaining poor storage conditions can induce bacterial growth and enterotoxin production [9].

For the detection of SE in food, the most commonly applied methods are those based on enzyme immunoassay (EIA), including enzyme-linked immunosorbent assay (ELISA) and enzyme-linked fluorescent assay (ELFA) [10,11] as well as reverse passive latex agglutination (RPLA) [12,13]. For economic reasons, antibodies directed against only certain SE types are available; therefore, these tests enable the detection of only five enterotoxin types: SEA-SEE, or in the case of RPLA, only four: SEA-SED [14].

According to the Centers for Disease Control and Prevention [15], staphylococcal enterotoxin poisoning can cause a short-term disease lasting from 1 to 3 days, but immunocompromised individuals, children or the elderly are more susceptible to a severe disease requiring intravenous treatment and hospital care. The name ‘staphylococcal toxins’ was correlated with their activity to induce vomitory reflexes [16]. Only the toxins that cause vomiting after being introduced to the digestive system can be referred to as staphylococcal enterotoxins (SE), while the toxins that exhibit no ability to induce a vomitory effect on primate models, or have not yet been tested for such properties, are referred to as staphylococcal-like enterotoxins (SEI) [16].

Currently, both enterotoxin groups are represented by 23 toxins [9,17]. These include five basic toxins, SEA, SEB, SEC, SED and SEE [7,18] (new toxins SEG, SEH, SEI, SER, SES, SET, as well as staphylococcal-like toxins such as SElJ, SElK, SElL, SElM, SElN, SElO, SElP, SElQ, SElU, SElU2, SElV, SElX [19,20,21,22,23,24]. The toxins with proven vomit-inducing ability include SEA to SEI, SER, SES, SEQ and SET. Several epidemiological studies have also shown that SEl genes, in particular the SEK, SEL, SEM, SEN and SEO genes, are often detected in strains isolated from food poisoning patients [25]. As some of these isolates do not possess any classical SE genes, these findings suggest that the recently described SEI are food poisoning agents and play an important role in the pathogenicity of staphylococci [26]. The toxic shock syndrome toxin-1 (TSST-1) also belongs to a family of toxins associated with SE and is capable of stimulating large populations of T cells containing a specific element Vβ in their T-cell receptors (TCR) [27].

Despite the homology between SE and SEI, the exact role of staphylococcal enterotoxin-like toxins is still unknown; however, the location of their genes on mobile genetic elements implies that they are part of virulence factors and serve a defensive role against the host’s immune system, and thus act against both adaptive and innate immunity [28,29].

This study aimed to assess the occurrence of genes responsible for the production of staphylococcal enterotoxins (SE), enterotoxin-like toxins (SEI) and toxic shock syndrome toxin-1 (TSST-1) in coagulase-negative staphylococci (CoNS) isolated from ready-to-eat food from bars and restaurants.

## 2. Results

Eighty five out of 118 isolates (72%) presented at least one gene encoding for enterotoxin production while 33/118 isolates (28%) were negative for all tested genes. The CoNS under study had genes encoding SEI more often than those encoding SE. Among the genes encoding SEI, the presence of the genes *selq* (23/26.7%), *sei* (18/20.9%), *ser* (17/19.8%), *selu* (12/14.0%) and *seh* (11/12.8%) was noted most frequently (Table 1). Among the genes encoding basic enterotoxins, the presence was noted of genes *sec* in 12 isolates (14%) belonging to the species *S. carnosus* (n = 2), *S. epidermidis* (n = 6), *S. warneri* (n = 3) and *S. haemolyticus* (n = 1), while the gene encoding the SED toxin was found in five isolates (5.8%) belonging to the species *S. saprophyticus* (n = 3), *S. pasteurii* (n = 1) and *S. simulans* (n = 1). The *sea*, *seb* and *see* genes were not detected in any of the strains studied (Table 1). None of these genes exhibited phenotypic expression by using mini Vidas and SET-RPLA tests. This means that despite the presence of strains with genes encoding SEA-SEE toxins, they did not show the ability to produce toxins on a laboratory scale. The ability to produce toxins is determined by many factors. Nevertheless, the test methods used are reference tests, therefore the lack of toxin production is caused by impaired expression rather than by a method error.

The study also examined the presence of exfoliative genes (*eta, etd*) as well as the toxic shock syndrome gene (*tsst-1*). The presence of the *tsst-1* gene encoding toxic shock syndrome toxin-1 (TSST-1) was confirmed in 27 (31.4%) CoNS strains belonging to the following species: *S. simulans* (n = 8), *S. carnosus* (n = 6), *S. epidermidis* (n = 3), *S. warneri* (n = 3), *S. xylosus* (n = 3), *S. saprophyticus* (n = 2), *S. pasteuri* (n = 1) *S. petrasii* subsp. *petrasii* (n = 1), *S. piscifermentas* (n = 1). Among the genes under study, which encoded exfoliative toxins, the presence of both genes at the same time was noted in two *S. pasteuri* strains (n = 2) and two *S. warneri* strains (n = 2). The *etd* gene occurred independently in *S. epidermidis* (n = 1), *S. lugdunensis* (n = 1) and *S. saprophyticus* (n = 1), while the *eta* gene occurred in *S. saprophyticus* (n = 2), *S. warneri* (n = 1) and *S. piscifermentas* (n = 1) strains (Table 1).

A total of 42 different toxin gene combinations were observed among the 85 toxin gene-positive CoNS isolates (Table 2). Ten strains had only the *tst-1* gene. The most common toxin gene combination was *seh, sec* which was present in four isolates and *selq, sei, ser, selu, selp* was present in three isolates. Sixteen isolates had both classical and newly described SAg genes. Two strains had only classical SAg genes.

The presence of toxin genes differed between individual species (Table 3). All isolates of *S. lentus* and *S. xylosus* were positive for toxin genes. Considering that the strains belonging to the species *S. warneri* display high pathogenic potential, it was not surprising that 92.9% of them had at least one gene coding for toxins. Furthermore, up to 4 out of 5 tested *S. carnosus* strains (considered non-pathogenic and used, among others, for the production of fermented food) also had toxin genes. A high percentage of isolates with SAgs genes were also found among *S. simulans* (88.9%) followed by *S. saprophyticus* (66.7%) and *S. epidermidis* (61.9%) (Table 1).

## 3. Discussion

The presence of enterotoxins produced by CoNS is not determined in routine food tests which can identify only the presence of five of them (i.e., SEA–SEE) among the 23 identified enterotoxins which are typical of *S. aureus.* In the EU, the detection of staphylococcal enterotoxins SEA-SEC is an obligatory procedure in the routine inspection of certain food products in which *S. aureus* has been identified at a level of at least 10^5^ cfu/g. Therefore, the presence of toxins produced by CoNS is not subject to control [30]. The results of the current study show that genes encoding the SEC toxin were found in CoNS belonging to the following species: *S. epidermidis, S. warneri, S. haemolyticus* and *S. carnosus*. While the occurrence of increased virulence in *S. epidermis*, *S. warneri* and *S. haemolyticus* is not surprising, the *S. carnosus* in food has so far been regarded as a non-pathogenic strain commonly used as starter cultures for the production of, for instance, fermented sausages [31]. Among the nine *S. carnosus* strains identified in this study, as many as four had at least one gene encoding such toxins as *seh*, *sec*, *tsst-1* or *selu*. This indicates the need for monitoring the possibility of toxin production by *S. carnosus* strains before their use as culture starters.

No compatibility between the presence of genes encoding basic toxins and the strains’ ability to produce toxins can be explained by gene mutation or the absence of regulatory genes necessary for expression inside the operon. Many studies also indicate that toxins are produced only under specific conditions determined by temperature, pH or appropriate medium properties. Although the studies conducted using the SET-RPLA and VIDAS tests were carried out in line with the manufacturer’s protocols, no ability of strains to produce toxins was noted. However, despite the lack of a toxin, the very fact of finding the presence of genes encoding them is worrying.

Although encoding staphylococcal enterotoxins on mobile genetic elements (MGE) poses a risk of their dissemination between species, including the pathogenic *S. aureus* strains, to a certain degree it also determines the gene arrangement in individual strains. SE and SEI can be carried on such MGEs as plasmids, prophages, transposons, pathogenicity islands and highly variable genomic vSa regions. The gene most frequently noted in the current study was (except the *tsst-1*) the *selq* gene. This gene, along with the *seb* and *selk*, is encoded by the genomic island vSa1 (SaPI3) [32]. The *selq* gene can also be carried on the φSa3m prophage [33]. Considering that the *selk* gene was found only in 10 CoNS, and the *seb* gene was found in none of the isolates, there is a possibility that the prophage was responsible for the presence of the *selq* gene in the studied isolates from ready-to-eat food.

Prophage also carry the *see*, *selk* and *selp* genes. One prophage is able to carry more than one gene encoding SE/SEI. SEA, SElK and SElQ are encoded together by one of two prophages, i.e., φSa3ms (Sa3, Fb, 255b) and φSa3mw (Sa3, Fb, 255a) [18,29,34,35]. In addition to chromosomally integrated prophages, small, low-chromium, linear/episomal and circular/plasmid extrachromosomal phages (ExPφs) are also found to be able to carry the *sei* and *selp* genes in staphylococcal strains [36].

The gene encoding the SEQ toxin was found in up to 23 strains belonging to the species *S. epidermidis* (7), *S. simulans* (5), *S. warneri* (5), *S. xylosus* (3), *S. haemolyticus* (1), *S. pasteuri* (1) and *S. saprophyticus* (1). This appears to be worrying because the results of a study conducted by Hu et al. [37] demonstrated that SEQ exhibits unusual stability during thermal processing and enzyme degradation as well as a significant vomit-inducing activity, which shows that the SEQ is a high-risk toxin in food poisoning.

Moreover, Johler et al. [38] provided evidence implying that enterotoxins, or a combination of the enterotoxins SEG, SEI, SEM, SEN and SEO, caused staphylococcal food poisoning. Two outbreaks recorded in the Swiss Federal Office of Public Health were analysed in which only strains carrying the *egc* cluster were isolated, including *seq*, *sei*, *sem*, *sen* and *seo* which are associated with typical staphylococcal food poisoning symptoms. The outbreaks were caused by the consumption of raw goat cheese and semi-hard goat cheese. These outbreaks provide further evidence that the newly described staphylococcal enterotoxins can cause staphylococcal food poisoning in humans.

The conducted study showed the insignificant percentage of genes encoding classic SE in CoNS strains isolated from ready-to-eat food, with a predominance of the SEI-encoding genes. Currently, it is the classic enterotoxins that are considered to be the main factor contributing to SFP outbreaks [9,39,40]. This situation is mainly due to the lack of sensitive methods for detecting non-classic toxins. However, the number of immunological tests for SEI, steadily increasing in recent decades, including SEG [41] SEH [42], SEI [43], SEK [44], SEM [45] and SEQ [37], enabled the demonstration that new enterotoxins can also be a possible cause of SFP outbreaks. Therefore, the presence of SEI-encoding genes in the CoNS genome can raise concerns about their involvement in SFP. In order to control staphylococcal food poisoning and to ensure food safety, the roles of both classic and new toxins need to be considered.

## 4. Materials and Methods

### 4.1. Isolation of Staphylococci Strains

Ready-to-eat food samples (n = 198) including sushi, salads, fresh squeeze juices, hamburgers, beef tartar, salmon tartar obtained from 11 randomly selected bars and restaurants in Olsztyn, Poland were investigated. Isolation of the strains was performed by methods described previously [46]. Briefly, food samples (10 g) were homogenised in 90 mL buffered peptone water (Merck, Germany), incubated overnight at 37 °C and streaked on selective plates containing Mannitol Salt Phenol-red Agar (Merck, Darmstadt, Germany). Mannitol (+) colonies were differentiated into coagulase-positive and coagulase-negative with a test detecting the production of a clumping factor (Staphylase Test Kit, Oxoid, Basingstoke, UK) and production of coagulase on the RPF medium (bioMérieux, Marcy l’Etoile, France) with rabbit blood plasma and fibrinogen.

### 4.2. Identification of CoNS by MALDI-TOF

After the initial phenotypic analysis, coagulase-negative strains were identified by MALDI-TOF using a VITEK MS instrument (bioMérieux, Marcy l’Etoile, France). Briefly, a portion of a bacterial colony (~1 μL) was spotted onto a MALDI sample plate, overlaid with 1 μL of a saturated solution of α-cyano-4-hydroxycinnamic acid in acetonitrile (28%), and then allowed to dry at room temperature. For each isolate, a mean spectrum was constructed with at least 50 m/z spectra profiles and used for the identification by comparison with the spectra contained in the Myla database (bioMérieux). Identification was defined as a 99–100% match to the species-specific *m*/*z* profiles in the database. *Escherichia coli* ATCC 8739 (bioMérieux, Marcy l’Etoile, France) was used as a standard for calibration and quality control.

### 4.3. Detection of Staphylococcal Superantigen Genes by Multiplex PCR

The presence of genes encoding enterotoxins, enterotoxin-like proteins, exfoliative toxins and TSST-1 in the investigated CoNS strains was determined by five multiplex PCR as described by [47,48,49,50]. All the primers were described in Table S1. The combinations of each reaction mixture (25 μL), consisted of 1 U AmpliTaq Gold DNA Polymerase with 1X PCR buffer (Applied Biosystems Inc., Foster City, CA, USA), 100 nM of dNTP mix (Applied Biosystems Inc.), 0.15 to 0.4 μM of each primer and 20–50 ng of template DNA. The multiplex PCR for SAg genes was carried out with the following thermal cycling conditions: an initial denaturation of DNA at 95 °C for 10 min was followed by 35 cycles of amplification (95 °C for 30 s, 55 °C for 45 s, and 72 °C for 60 s), ending with a final extension at 72 °C for 10 min. The amplified PCR products were visualised by standard gel electrophoresis in a 1.5% agarose gel stained by ethidium bromide (5 μg/mL). The gels were photographed under ultraviolet light using the G-Box system (Syngene, Cambridge, UK) and analysis using GeneTools software (Syngene, UK). Control strains included *S. aureus* A920210 (*eta*) [51], *S. aureus* Col (*seb, selk*, *selq*) [51], *S. aureus* FRI1151m (*sed, selj, ser*) [50], *S. aureus* FRI137 (*sec, seh, sell, selu*) [51], *S. aureus* FRI913 (*sea, sec, see, selk, sell, selq, tsst-1*) [51], *S. aureus* N315 (*sec, seg, sei, sell, selm, seln, selo, selp, tsst-1*) [52], *S. aureus* TY114 (*etd*) [51], *S. aureus* 8325-4 (no SAgs genes) [50]. Amplification of non-template controls was also included in each analysis to determine if DNA contamination had occurred.

### 4.4. Enterotoxin Detection

Enterotoxins produced by CoNS in culture media was detected by using mini-Vidas (bioMerieux) and SET-RPLA (Oxoid). First, the mini-Vidas (bioMerieux) test was performed to assess the presence of SEs. This method presents a sensitivity of 1 ng/g and allows the detection of 7 SEs: A, B, C1, C2, C3, D, and E [11]. However, as it uses antienterotoxins polyclonal antibodies, it does not allow the discrimination of the type of SE produced. However, SET-RPLA uses antienterotoxins monoclonal antibodies and may detect SEs A to D with a sensitivity of 0.25 ng/mL [53]. Therefore, this test was used to determine the type of SE produced. The results of both methods were expressed as presence or absence of SE by CoNS strains.

### 4.5. Detection of Staphylococcal Enterotoxins A-D by Staph Enterotoxin II Test

The enzyme-linked fluorescent assay (ELFA) using the automated VIDAS instrument was used for the specific detection of Staphylococcal enterotoxins (Staph enterotoxin II, SET 2, bioMerieux) according to the instructions of the manufacturer. One isolated colony of every strain was cultured in BHI broth for 24 h at 37 °C. The culture was centrifuged at 7000 g for 10 min at 4 °C and 500 mL of the supernatant was then added to the initial VIDAS strip wells and further analysed by the automated method. In this test, complementary monoclonal and polyclonal antibodies directed to the 5 different staphylococcal enterotoxins SEA, SEB, SEC, SED and SEE are used for the capture and detection process without distinguishing individual toxins.

### 4.6. Enterotoxin Identification by ReSversed Passive Latex Agglutination (RPLA)

SET-RPLA Toxin Detection Kit (Oxoid, UK) was used to detect enterotoxigenic strains. One isolated colony of every strain was cultured in BHI broth at 37 °C overnight. Twenty-five microliters of culture were placed in V-shaped 96-well plates. After that, 25 μL of latex-sensitised particles solution was added and plates were kept at room temperature for 24 h without agitation. Agglutination of latex particles observed against a black bottom was considered a positive result.

## 5. Conclusions

The presence of SAg genes in CoNS confirms the possible role of these bacteria in food intoxication. The occurrence of CoNS in food should not be ignored nor should their pathogenic potential be considered as insignificant, rather safety measures should be taken to reduce or totally eliminate their occurrence in foods

## Figures and Tables

**Table 1:** Results of toxin genes presence depending on the species.

Species	No. of Isolates	% of SAgs Positive *	Number (%) of Toxin Gene–Positive Isolates																					
			*sea*	*seb*	*sec*	*sed*	*see*	*Seg*	*seh*	*się*	*selj*	*selk*	*sell*	*selm*	*seln*	*selo*	*selp*	*selq*	*selu*	*ser*	*eta*	*etd*	*tsst-1*	
*S. epidermidis*	21	13 (61.9)			6 (28.6)			1 (4.8)	5 (23.8)	4 (19)	1 (4.8)		4 (19)		1 (4.8)	2 (9.5)	1 (4.8)	7 (33.3)	2 (9.5)	4 (19)		1 (4.8)	7 (33.3)	
*S. warneri*	14	13 (92.9)			3 (2.4)			5 (35.7)	3 (21.4)	3 (21.4)	3 (21.4)				3 (2.4)		3 (2.4)	5 (35.7)	3 (21.4)	3 (21.4)	2 (14.3)	1 (7.1)	3 (21.4)	
*S. carnosus*	9	4 (44.4)			2 (22.2)				2 (22.2)										1 (11.1)				1 (11.1)	
*S. simulans*	9	8 (88.9)				1 (11.1)				1 (11.1)			1 (11.1)					5 (55.6)		2 (22.2)			7 (8.1)	
*S. xylosus*	8	8 (100)								3 (37.5)					1 (12.5)		2 (25)	3 (37.5)	3 (37.5)	4 (50)			3 (37.5)	
*S. saprophyticus*	6	4 (66.7)				3 (50)				1 (16.7)		2 (33.3)		1 (16.7)	2 (33.3)	1 (16.7)	1 (16.7)	1 (16.7)		1 (16.7)	2 (33.3)	1 (16.7)	2 (33.3)	
*S. pasteuri*	5	3 (60.0)				1 (20)				2 (40)	1 (20)	1 (20)			1 (20)		1 (20)	1 (20)	1 (20)	2 (40)	1 (20)	1 (20)	1 (20)	
*S. heamolyticus*	4	1 (25.0)			1 (25)			1 (25)	1 (25)				1 (25)			1 (25)		1 (25)					1 (25)	
*S. petrasii subsp. Petrasii*	4	2 (50.0)						1 (25)		1 (25)							1 (25)						1 (25)	
*S. lentus*	2	2 (100)						1 (50)		2 (100)							1 (50)		1 (50)	1 (50)				
*S. piscifermentas*	2	1 (50.0)								1 (50)											1 (50)		1 (50)	
*S. lugdenensis*	1	1 (100.0)																	1 (100)			1 (100)		
Total	85	60 (69.7)	0	0	12 (14)	5 (5.8)	0	9 (10.5)	11 (12.8)	18 (20.9)	5 (5.8)	3 (3.5)	6 (7.0)	1 (1.2)	8 (9.3)	4 (4.7)	10 (11.6)	23 (26.7)	12 (14.0)	17 (19.8)	6 (7.0)	5 (5.8)	27 (31.4)	

* strains positive for at least one toxin gene.

**Table 2:** The profiles of enterotoxin genes in Staphylococcus spp. isolated from ready-to-eat food.

	Toxin Genes Occurence	Number (%) of Strians		Number of Genes
1	*tst-1*	10	(11.63)	1
2	*etd*	1	(1.16)	
3	*sec*	1	(1.16)	
4	*sed*	1	(1.16)	
5	*sei*	1	(1.16)	
6	*selu*	1	(1.16)	
7	*edt, selu*	1	(1.16)	2
8	*seg, ser*	1	(1.16)	
9	*sei, ser*	1	(1.16)	
10	*seln, selp*	1	(1.16)	
11	*tst-1, eta*	1	(1.16)	
12	*tst-1, selk*	1	(1.16)	
13	*seh, sec*	4	(4.65)	
14	*tst-1, selq*	2	(2.33)	
15	*seg, sei, selp*	1	(1.16)	3
16	*seln, sei, ser*	1	(1.16)	
17	*seln, selq, selk*	1	(1.16)	
18	*selq, sei, ser*	1	(1.16)	
19	*selq, ser, selu*	1	(1.16)	
20	*tst-1, sed, eta,*	1	(1.16)	
21	*tst-1, sei, ser*	1	(1.16)	
22	*tst-1, sell, selq*	1	(1.16)	
23	*tst-1, eta, sei*	1	(1.16)	
24	*sei, selu, selp*	2	(2.33)	
25	*tst-1, selq, ser*	1	(1.16)	
26	*etd, eta, selu, selp*	1	(1.16)	4
27	*seg, selq, selj, sei*	1	(1.16)	
28	*seh, sec, tst-1, selq*	1	(1.16)	
29	*seln, seg, selq, selj*	1	(1.16)	
30	*seln, seg, selq, selu*	1	(1.16)	
31	*seln, selq, sei, ser*	1	(1.16)	
32	*selq, ser, selu, selp*	1	(1.16)	
33	*tst-1, sed, eta, selk*	1	(1.16)	
34	*seh, sec, tst-1, sell, selq*	1	(1.16)	5
35	*seh, sec, tst-1, sell, ser*	1	(1.16)	
36	*selq, sei, ser, selu, selp*	3	(3.49)	
37	*seh, sec, tst-1, sell, selo, selq*	2	(2.33)	6
38	*seh, sec, tst-1, sell, selo, seg, selq*	1	(1.16)	7
39	*seln, seg, selq, selj, sei, ser, selp*	1	(1.16)	
40	*seln, seg, selq, selj, sei, ser, selu*	1	(1.16)	
41	*tst-1, sed, etd, eta, selk, sei, sell*	1	(1.16)	
42	*sed, etd, selk, selm, selo, seln, selq, sei, ser*	1	(1.16)	9
43	Negative for toxin genes	27	(31.40)	0

**Table 3:** Presence of toxin genes in Staphylococcus spp. isolated from ready-to-eat food.

Species	Combination of SAg genes	Number (%) of Isolates
*S. carnosus* (n = 9)	*seh, sec*	2 (22.2)
	*tst-1*	1 (11.1)
	*selu*	1 (11.1)
	none	5 (55.6)
*S. epidermidis* (n = 21)	*sei*	1 (4.8)
	*etd*	1 (4.8)
	*sec*	1 (4.8)
	*seh, sec, tst-1, sell, selo, selq*	2 (9.5)
	*seh, sec, tst-1, sell, selq*	1 (4.8)
	*seh, sec, tst-1, sell, ser*	1 (4.8)
	*seh, sec, tst-1, selq*	1 (4.8)
	*seln, seg, selq, selj, sei, ser, selu*	1 (4.8)
	*selq, sei, ser*	1 (4.8)
	*selq, sei, ser, selu, selp*	1 (4.8)
	*tst-1*	2 (9.5)
	none	8 (38.1)
*S. haemolyticus* (n = 4)	*seh, sec, tst-1, sell, selo, seg, selq*	1 (25)
	none	3 (75)
*S. lentus* (n = 2)	*sei, selu, selp*	1 (50)
	*seln, sei, ser*	1 (50)
*S. lugdenensis* (n = 2)	*edt, selu*	1 (50)
	none	1 (50)
*S. pasteuri* (n = 5)	*seln, selq, selk*	1 (20)
	*tst-1, sed, etd, eta, selk, sei, sell*	1 (20)
	*selq, sei, ser, selu, selp*	1 (20)
	none	2 (40)
*S. petrasii subsp. petrasii* (n = 4)	*tst-1*	1 (25)
	*seg, sei, selp*	1 (25)
	none	2 (50)
*S. piscifermrntans* (n = 2)	*tst-1, eta, sei*	1 (50)
	none	1 (50)
*S. saprophyticus* (n = 6)	*sed, etd, selk, selm, selo, seln, selq, sei, ser*	1 (16.7)
	*seln, selp*	1 (16.7)
	*tst-1, sed, eta, selk*	1 (16.7)
	*tst-1, sed, eta*	1 (16.7)
	none	2 (33.3)
*S. simulans* (n = 9)	*sed*	1 (11.1)
	*tst-1*	1 (11.1)
	*tst-1, sei, ser*	1 (11.1)
	*tst-1, selk*	1 (11.1)
	*tst-1, sell, selq*	1 (11.1)
	*tst-1, selq*	2 (22.2)
	*tst-1, selq, ser*	1 (11.1)
	none	1 (11.1)
*S. warneri* (n = 14)	*etd, eta, selu, selp*	1 (7.1)
	*seg, selq, selj, sei*	1 (7.1)
	*seg, ser*	1 (7.1)
	*seh, sec*	3 (21.4)
	*seln, seg, selq, selj*	1 (7.1)
	*seln, seg, selq, selj, sei, ser, selp*	1 (7.1)
	*seln, seg, selq, selu*	1 (7.1)
	*selq, sei, ser, selu, selp*	1 (7.1)
	*tst-1*	2 (14.3)
	*tst-1, eta*	1 (7.1)
	none	1 (7.1)
*S. xylosus* (n = 8)	*sei, selu, selp*	3 (37.5)
	*sei, ser*	1 (12.5)
	*seln, selq, sei, ser*	1 (12.5)
	*selq, ser, selu*	1 (12.5)
	*selq, ser, selu, selp*	1 (12.5)
	*tst-1*	1 (12.5)

## Supplementary Materials

The following are available online at https://www.mdpi.com/2076-0817/9/9/734/s1, Table S1: List of primer sequences used in PCR reactions.
