# Supplementary material for: Enterotoxigenic Potential of Coagulase-Negative Staphylococci from Ready-to-Eat Food
Source: Pathogens. 2020 Sep 6;9(9):734. doi: 10.3390/pathogens9090734 (PMC7559265; doi:10.3390/pathogens9090734)
Supplement: Supplementary file 1 [file pathogens-09-00734-s001.pdf]

Supplementary materials:

Table S1. List of primer sequences used in PCR reactions.

	Genes	Primers	Sequence (5′ → 3′)	Product size (bp)	References
**Multiplex I**	*sea*	sea-1	GAA AAA AGT CTG AAT TGC AGG GAA CA	560	[1]
		sea-2	CAA ATA AAT CGT AAT TAA CCG AAG GTT C		
	seh	seh-1	CAA TCA CAT CAT ATG CGA AAG CAG	376	[1]
		she-2	CAT CTA CCC AAA CAT TAG CAC C		
	sec	sec-1	CTT GTA TGT ATG GAG GAA TAA CAA AAC ATG	275	[1]
		sec-2	CAT ATC ATA CCA AAA AGT ATT GCC GT		
	tst-1	tst-1	TTC ACT ATT TGT AAA AGT GTC AGA CCC ACT	180	[1]
		tst-2	TAC TAA TGA ATT TTT TTA TCG TAA GCC CTT		
**Multiplex II**	*sed*	sed-1	GAA TTA AGT AGT ACC GCG CTA AAT AAT ATG	492	[1]
		sed-2	GCT GTA TTT TTC CTC CGA GAG T		
	etd	etd-1	CAA ACT ATC ATG TAT CAA GGA TGG	358	[2]
		etd-2	CCA GAA TTT CCC GAC TCA G		
	eta	eta-1	ACT GTA GGA GCT AGT GCA TTT GT	190	[1]
		eta-2	TGG ATA CTT TTG TCT ATC TTT TTC ATC AAC		
	selk	sek-1	ATG CCA GCG CTC AAG GC	134	[3]
		sek-2	AGA TTC ATT TGA AAA TTG TAG TTG ATT AGC T		
		sek-3	TGC CAG CGC TCA AGG TG		
**Multiplex III**	*see*	see-1	CAA AGA AAT GCT TTA AGC AAT CTT AGG C	482	[1]
		see-2	CAC CTT ACC GCC AAA GCT G		
	seb	seb-1	ATT CTA TTA AGG ACA CTA AGT TAG GGA	404	[1]
		seb-2	ATC CCG TTT CAT AAG GCG AGT		
	selm	sem-1	CTA TTA ATC TTT GGG TTA ATG GAG AAC	326	[1]
		sem-2	TTC AGT TTC GAC AGT TTT GTT GTC AT		
	sell	sel-1	GCG ATG TAG GTC CAG GAA AC	234	[3]
		sel-2	CAT ATA TAG TAC GAG AGT TAG AAC CAT A		
	selo	seo-1	AGT TTG TGT AAG AAG TCA AGT GTA GA	180	[1]
		seo-2	ATC TTT AAA TTC AGC AGA TAT TCC ATC TAA C		
**Multiplex IV**	*seln*	sen-1	CGT GGC AAT TAG ACG AGT C	474	[3]
		sen-2	GAT TGA TYT TGA TGA TTA TKA G		
	seg	seg-1	TCT CCA CCT GTT GAA GG	323	[3]
		seg-2	AAG TGA TTG TCT ATT GTC G		
	selq	seq-1	ACC TGA AAA GCT TCA AGG A	204	[3]
		seq-2	CGC CAA CGT AAT TCC AC		
	selj	sej-1	TCA GAA CTG TTG TTC CGC TAG	138	[3]
		sej-2	GAA TTT TAC CAY CAA AGG TAC		
**Multiplex V**	*sei*	sei-1	CTY GAA TTT TCA ACM GGT AC	461	[3]
		sei-2	AGG CAG TCC ATC TCC TG		
	ser	ser-1	AGC GGT AAT AGC AGA AAA TG	363	[3]
		ser-2	TCT TGT ACC GTA ACC GTT TT		
	selu	seu-1	AAT GGC TCT AAA ATT GAT GG	215	[3]
		seu-2	ATT TGA TTT CCA TCA TGC TC		
	selp	sep-1	GAA TTG CAG GGA ACT GCT	182	[3]
		sep-2	GGC GGT GTC TTT TGA AC		

References

1. Jarraud, S.; Mougel, C.; Thioulouse, J.; Lina, G.; Meugnier, H.; Forey, F.; Nesme, X.; Etienne, J.; Vandenesch, F. Relationships between *Staphylococcus aureus* genetic background, virulence factors, *agr* groups (alleles), and human disease. *Infect. Immun.* **2002**, *70*, 631-641.
2. Zhang, S.; Iandolo, J.J.; Stewart, G.C. The enterotoxin D plasmid of *Staphylococcus aureus* encodes a second eneterotoxin determination (*sej*). *FEMS Microbiol. Lett.* **1998**, *168*, 227–233.
3. Holtfreter, S.; Grumann, D.; Schmudde, M., Nguyen, H.T.T.; Eichler, P.; Strommenger, B.; Kopron, K.; Kolata, J.; Giedrys-Kalemba, S.; Steinmetz, I.; Witte, W.; Bröker**,** B. M. Clonal distribution of superantigen genes in clinical *Staphylococcus aureus* isolates. *J. Clin. Microbiol.* **2007**, *45***,** 2669-2680.
